# Pancreatic cancer risk and survival in patients with Lynch syndrome: a nationwide Dutch cohort study

**DOI:** 10.1016/j.eclinm.2026.103755

**Published:** 2026-01-12

**Authors:** Aleksander M. Bogdanski, Derk C.F. Klatte, Bert A. Bonsing, Lodewijk A.A. Brosens, Evelien Dekker, Lydia G. van der Geest, Joep E.G. Ijspeert, Jan J. Koornstra, Mariëtte C.A. van Kouwen, Alexandra M.J. Langers, Maartje Nielsen, Dewkoemar Ramsoekh, Manon C. Spaander, Wouter H. de Vos Tot Nederveen Cappel, Jeanin E. Van Hooft, Monique E. van Leerdam, Dutch Pancreatic Cancer Group, A.A. Tanis, A.Y. Thijssen, W.R. ten Hove, J. Sint Nicolaas, F. Voogd, N.C. Talstra, M.J. van Heerde, C.C.G. van Enckevort, E.J. Schoon, C. Postma, A. Geraedts, F. ter Borg, J. Geesing, M.G.F. van Lier, W. Hazen, M.L. Hazen, M.W. Mundt, I. Leeuwenburgh, G.W. Erkelens, M. Kerkhof, J.J. Keller, M.H.M.G. Houben, J.P. De Filippi, T.J. Tang, C. Verveer, J.S. Terhaar sive Droste, M.W. van den Berg, P.J. Bus, J.J.L. Haans, W.J. Thijs, M.L. Verhulst, L.G. Capelle, S. Corporaal, M. Bigirwamungu-Bargeman, A.M. Zonneveld, A.M. van Berkel, W.E. Boertien, A.U.G. van Lent, S.A. Mulder, F.A. Oort, M.I.E. Appels, R. Andriessen, W.A. Marsman, S. de Kort, A. Al-Toma, M. van Boekel, M.E. Smits, P.E.P. Dekkers, T. Kuiper, J.E. van Rooij, R. Meiland, L. van Vlerken, H. Aktas, E. Rondagh, Collaborative investigators from the Dutch Foundation for Detection of Hereditary Tumors

**Affiliations:** a Department of Gastroenterology and Hepatology, Leiden University Medical Center, Leiden, the Netherlands; b Department of Surgery, Leiden University Medical Center, Leiden, the Netherlands; c Department of Pathology, University Medical Center Utrecht, Utrecht University, Utrecht, the Netherlands; d Department of Gastroenterology and Hepatology, Amsterdam University Medical Center, Amsterdam, the Netherlands; e Department of Research and Development, Netherlands Comprehensive Cancer Organization (IKNL), Utrecht, the Netherlands; f Department of Gastrointestinal Oncology, Netherlands Cancer Institute, Amsterdam, the Netherlands; g Department of Gastroenterology and Hepatology, University Medical Center Groningen, Groningen, the Netherlands; h Department Gastroenterology and Hepatology, Radboud University Medical Centre Nijmegen, Nijmegen, the Netherlands; i Department of Clinical Genetics, Leiden University Medical Center, Leiden, the Netherlands; j Department of Gastroenterology and Hepatology, Erasmus University Medical Center, Rotterdam, the Netherlands; k Department of Gastroenterology and Hepatology, Isala Zwolle, Zwolle, the Netherlands

**Keywords:** Pancreas cancer, Lynch syndrome, Surveillance, Cumulative incidence

## Abstract

**Background:**

Individuals with Lynch syndrome (LS) are advised to undergo pancreatic ductal adenocarcinoma (PDAC) surveillance if their lifetime risk is ≥5%, however, evidence is limited. This study quantifies lifetime risk and survival of three cancers relevant to PDAC surveillance, including PDAC, ampullary carcinoma (AC) and distal cholangiocarcinoma (dCC), to evaluate whether surveillance is justified.

**Methods:**

This retrospective nationwide Dutch cohort study included individuals with LS pathogenic variants (PVs) in *MLH1*, *MSH2*, *MSH6*, *PMS2* or *EpCAM* (identified between 1985 and 2024) and compared them to sporadic cases from the general population (diagnosed between 2000 and 2022). Cumulative incidence (CI) of PDAC, AC and dCC was estimated using Fine-and-Gray models for LS and a CI formula for sporadic cases. Relative risks (RRs) were calculated by comparing CIs. Survival of the cancers was compared between both cohorts using 1:10 matched analyses by age at diagnosis, sex, stage, and year of diagnosis.

**Findings:**

In total, 2605 individuals with LS were included (median age 63.9 years; IQR 53.7–74.0), of whom 1515 (58.2%) were female. PVs were identified in *MLH1* (723, 27.8%), *MSH2* (895, 34.4%), *MSH6* (731, 28.1%), *PMS2* (233, 8.9%) and *EpCAM* (23, 0.9%). By age 75, the combined CI of PDAC, AC and dCC was 3.0% (95% CIs, 1.5–5.8) in *MLH1*, 3.4% (95% CIs, 2.0–5.8) in *MSH2/EpCAM*, 1.0% (95% CIs, 0.3–2.7) in *MSH6* and 0% in *PMS2*. No familial-clustering of cancers was observed. Matched survival did not differ between PDACs in LS and sporadic cases. In contrast, survival was better for AC in LS (34.3 months; 95% CIs, 3.2–Inf) compared to sporadic cases (15.5 months; 95% CIs, 9.9–19.3).

**Interpretation:**

In LS, the combined lifetime risk of PDAC, AC and dCC ranged from 0 to 3.4% across different genes, remaining below the 5% risk threshold for PDAC surveillance. Additionally, having an affected relative did not appear to increase risk. These findings suggest that current surveillance recommendations for individuals with LS should be re-evaluated.

**Funding:**

Lynch-Polyposis.

**Research in context**

**Evidence before this study**

We searched PubMed without language or date restrictions through 17 November 2025 using the terms (pancreas cancer OR pancreatic cancer OR pancreas carcinom∗ OR pancreatic carcinom∗ OR PDAC OR pancreatic ductal adenocarcinom∗) AND (Lynch syndrome OR LS). The search identified four studies reporting cumulative pancreatic ductal adenocarcinoma (PDAC) incidence in Lynch syndrome, with estimates ranging from approximately 0.4%–3.7% by age 70. These studies were heterogeneous in design and in their methods of case ascertainment. Moreover, none of the studies were designed with focus on PDAC surveillance. A recent study published in October 2025 examined cholangiocarcinomas, but did not specifically assess distal cholangiocarcinomas (dCC) or ampullary carcinomas (AC). Consequently, periampullary tumors that could be detected by PDAC surveillance were not evaluated.

Additionally, the prior reports provided no information on familial clustering (first-degree relative PDAC risk), a key criterion used by guideline panels when suggesting surveillance for individuals with Lynch syndrome. Lastly, two of the four studies date from around 2010. Over the years, clinical management and genetic testing practices in individuals with Lynch syndrome have changed, and these developments could alter observed extracolonic cancer risks. As a result, older estimates may have limited relevance for current practice. Taken together, the existing literature is limited and lacks the family-history and periampullary cancer data required to inform PDAC surveillance guidelines.

**Added value of this study**

To our knowledge, this is the first nationwide study to estimate the cumulative incidence of PDAC, AC, and dCC in combination. By age 75, the cumulative incidence was 3.0% for *MLH1* carriers, 3.4% for *MSH2/EpCAM*, 1.0% for *MSH6* and 0% for *PMS2*. For none of the LS-associated genes did the cumulative incidence of PDAC by age 75 exceed the 5% threshold suggested by guidelines for initiating PDAC surveillance, even when AC and dCC were included. Furthermore, in our cohort, having one affected first-degree relative was not associated with an increased risk of PDAC. These nationwide data provide evidence directly relevant to informing future recommendations for PDAC surveillance in Lynch syndrome.

**Implications of all the available evidence**

When all available evidence is considered, routine PDAC surveillance for Lynch syndrome carriers on the basis of a single affected first-degree relative is not warranted when evaluated against the 5% lifetime-risk threshold. Future guidelines should therefore re-evaluate this advice.

## Introduction

Lynch syndrome (LS) is an autosomal dominant hereditary cancer predisposition syndrome with an estimated prevalence of 1 in 279 individuals at birth.^1^ The condition is caused by a pathogenic variant (PV) in one of the four mismatch repair (MMR) genes: *MLH1*, *MSH2*, *MSH6*, *PMS2* or by epigenetic silencing of *MSH2* through deletions in *EpCAM*.^1^ These variants compromise the DNA MMR system, resulting in an increased accumulation of replication errors and, consequently, a substantially elevated risk of cancer.^1^ LS is primarily associated with an increased lifetime risk of colorectal and endometrial cancer, for which surveillance strategies such as regular colonoscopies, gynecological surveillance and symptom awareness education are recommended.^2^ Additionally, LS has been associated with an increased risk of several other forms of cancer, including pancreatic ductal adenocarcinoma (PDAC).^3^, ^4^, ^5^ Despite the existence of PDAC surveillance programs, the relevance of including LS individuals in these protocols remain subjects of debate.

According to the International Cancer of the Pancreas Screening (CAPS) Consortium, individuals are considered eligible for PDAC surveillance if they have a minimum lifetime risk of 5%.^6^ A careful selection of these individuals is necessary, as participation in surveillance also has potential harms, such as overtreatment and psychosocial burden.^7^ Currently, PDAC surveillance is advised for LS carriers with a first-degree relative affected by PDAC.^6^ Nonetheless, the evidence supporting their PDAC risk exceeding the threshold, as established by CAPS, is limited.^8^ There are few studies on cumulative incidence of PDAC in LS and they report variable estimates ranging from 0.4% to 3.7% by the age of 70 years.^3^, ^4^, ^5^ This variability in risks makes it difficult to develop clear guidelines for these individuals. Furthermore, the cancer risk, including the risk of PDAC varies among different LS-associated PVs.^3^^,^^4^ An international cohort study reported a cumulative incidence of PDAC at age 70 of 3.9% for *MLH1*, 0.5% for *MSH2*, 1.4% for *MSH6* and 0% for *PMS2* PVs.^5^ These findings suggest that PDAC risk is not uniform across the different genes involved in individuals with LS and should be considered when developing surveillance guidelines.

In addition to PDAC, LS carriers are at an increased risk of developing other upper gastrointestinal malignancies, including ampullary carcinoma (AC) and distal cholangiocarcinoma (dCC).^5^ These malignancies are anatomically adjacent to the pancreas and lie within the field of view of surveillance modalities such as EUS or MRI. Similar to PDAC, both AC and dCC are associated with poor prognoses.^5^ The overall five-year survival for AC ranges from 4% to 45%, depending on the stage at diagnosis, while for dCC, it is approximately 11%.^9^^,^^10^ Given their anatomical proximity and poor outcomes, it is of interest to consider AC and dCC when evaluating the potential added value of PDAC surveillance.

Beyond the risk estimates, data concerning survival outcomes for LS patients diagnosed with PDAC, AC or dCC remains limited.^5^ Tumors in LS are typically characterized by microsatellite instability (MSI)/mismatch repair deficiency (MMRd), which renders them more responsive to immune checkpoint inhibitors and leads to improved prognoses relative to sporadic cases.^11^^,^^12^

To address the current knowledge gap and inform clinical decision-making, robust population-level estimates of pancreato-biliary cancer risk and survival among individuals with LS are needed. Therefore, using a nationwide dataset, we aim to quantify the lifetime risk and survival outcomes of PDAC, AC and dCC in this population.

## Methods

### Study design

This is a retrospective analysis that uses prospectively collected data from the national Dutch registry of families with risk of hereditary cancer, known as the Foundation for the Detection of Hereditary Tumors (StOET; https://www.stoet.nl/). Further detailed in Vasen et al.^13^ Individuals with LS are asked to register at the StOET by their healthcare professional. Informed consent for the use of pseudo-anonymized data in research was obtained from subjects upon their registration with StOET. The study received approval from the following entities: the institutional review board (IRB) of Leiden University Medical Center (reference: 2022050), the scientific committee of the Dutch Pancreatic Cancer Group (reference: 2022-12), the Dutch Nationwide Pathology Databank (Palga; reference: lzv2023-72) and the Privacy Review Board of the Netherlands Cancer Registry (NCR; reference: K22.386).

### Exposed cohort

The exposed cohort consists of individuals (≥18) with a confirmed or obligate PV in one of the following genes: *MLH1, MSH2, PMS2, MSH6* or by epigenetic silencing of *MSH2* through deletions in *EpCAM*. The subjects of this study were identified in the StOET registry from 1985 to 2024 and linked with the Palga. The StOET provided demographic data and data about clinically diagnosed PDACs without histological confirmation. Palga provided information on tumor characteristics, including stage, follow-up (last available pathology) and MSI/MMRd status for all histologically confirmed PDACs. Given that PDAC is frequently diagnosed at an advanced stage, at which point histological confirmation is not always pursued due to its limited impact on treatment decisions, both cases with a high clinical suspicion based on imaging and cases with histological confirmation were included to ensure an accurate estimation of the cumulative PDAC incidence. Data concerning survival status was obtained from the Center Bureau for Genealogy (CBG; https://cbg.nl/) via the national register of deceased persons. Information on cause of death was not available.

### Control cohort

To compare the cumulative incidence of PDAC, AC and dCC in individuals with LS to sporadic PDAC cases, data were obtained from the NCR (https://iknl.nl/en/NCR) and Statistics Netherlands (CBS; https://www.cbs.nl/). The number of cancer cases was derived from the NCR, while the age-specific number of individuals at risk per one-year age bracket was retrieved from the CBS. The age-specific population counts were derived from data collected on a yearly basis in January. The data from both sources covered the period from 2000 to 2022.

For the survival analyses, a dataset of all PDAC, AC and dCC cases diagnosed in the Netherlands was obtained from the NCR, including both histologically confirmed and clinically diagnosed cases.

### Definitions

It is important to acknowledge the rarity of PDAC, AC and dCC development in individuals under the age of 18. Consequently, the follow-up period began at age 18 and persisted until the diagnosis of one of the specified cancers, death, lost to follow up or the conclusion of follow up in July 2024. Cancer staging was conducted according to the 8th edition of the American Joint Committee on Cancer staging manual.^14^ As it is sometimes difficult to distinguish between PDAC and other cancers of the periampullary region, even with pathology, cases of uncertain origin were categorized as ‘suspected PDAC’ cases and were analyzed separately.

### Statistical analysis

A Fine-and-Gray subdistribution hazard model was used to estimate the cumulative incidence of PDAC, AC and dCC at ages 70, 75 and 80, adjusting for death as a competing risk.^15^ Cumulative incidence estimates were calculated for the entire LS cohort, as well as for each PV subgroup: *MLH1*, *MSH2*, *MSH6*, and *PMS2*. PVs in *EpCAM* were incorporated into the *MSH2* group, since *EpCAM* variants lead to epigenetic silencing of *MSH2*. To correct for ascertainment bias, individuals whose first clinic visit was prompted by a PDAC, AC or dCC diagnosis were excluded. The 95% confidence intervals (CIs) were transformed to the logit scale to ensure their validity for proportions and prevent negative values. In addition to cumulative incidence estimates for PDAC, AC and dCC, a sensitivity analysis was conducted in which PDAC cases were combined with suspected PDAC cases. Moreover, the cumulative incidence was calculated for all cancer types combined ((suspected) PDAC, AC and dCC).

The cumulative incidences observed in the LS cohort were compared with sporadic cases in the general population. Since the general population is a dynamic cohort for which only incidence rates can be calculated, person-time was approximated by dividing the population into one-year age groups and assuming a steady-state population within each interval.^16^ To account for individuals who developed PDAC, AC or dCC, it was assumed that these cancers developed halfway through the year. Consequently, a subtraction of 0.5 person-years was applied to the corresponding age group. Given that the cumulative incidence over short intervals can be estimated by multiplying the incidence rate by the duration of follow-up (one-year), the incidence rate for each age group was used to calculate the one-year risk for that specific age bracket. Cumulative incidence estimates up to ages 70, 75 and 80 were obtained by using the following formula *Cumulative incidence= 1 - exp[-∑(IRᵢ × tᵢ)]*. The RRs of PDAC, AC and dCC in individuals with LS were estimated by dividing the cumulative incidence in the LS cohort by that of sporadic cases.

Cancer cases from the LS cohort were matched to sporadic cases for comparison based on age at diagnosis, sex, cancer stage and year of diagnosis. The nearest neighbor matching was used, aiming for a 1:10 matching ratio and a standardized mean difference of less than 0.25. The two- and five-year survival rates for PDAC, AC and dCC were calculated, provided that sufficient case numbers were available. Kaplan–Meier survival analysis was used to estimate the survival rates and the log-rank test compared outcomes between the LS-associated and sporadic cancers.

Finally, we acknowledge the possibility that PDAC, AC and dCC cases from the LS-cohort are also present in the general population cohort. Due to limitations related to confidentiality, it was not possible to identify and exclude these cases from the general population group. However, given the substantial number of controls in the general population cohort, approximately 40,000, we anticipate that any potential overlap will have a minimal impact, particularly as matching was performed with multiple controls per case. All statistical analyses were carried out using the R project 4.4.2 and statistical significance was defined as a two-tailed p-value ≤0.05.

### Role of the funding source

The funder of the study had no role in study design, data collection, data analysis, data interpretation or writing of the report.

## Results

A total of 2605 individuals with genetically confirmed or obligate LS were included. The median age at the end of the study period was 63.9 years (IQR 53.7–74.0) and 1515 (58.2%) of the subjects were females. In the exposed cohort, 723 (27.8%) carried a PV in *MLH1*, 895 (34.4%) in *MSH2*, 731 (28.1%) in *MSH6*, 233 (8.9%) in *PMS2* and 23 (0.9%) in *EpCAM.* No individuals were excluded due to ascertainment bias, as this was not present in our cohort. For an overview of the baseline characteristics, refer to Table 1.

**Table 1:** Baseline characteristics of individuals with Lynch syndrome.

	Lynch syndrome cohort (N = 2605)
**Age**, median (IQR)	63.9 (53.7–74.0)
**Sex**, female, n (%)	1515 (58.2)
**Gene variant**, n (%)	
**MLH1**^a^	723 (27.8)
Age, median (IQR)	64.9 (54.2–74.5)
Female, n (%)	443 (61.3)
**MSH2**	895 (34.4)
Age, median (IQR)	62.7 (53.5–71.9)
Female, n (%)	487 (54.4)
**MSH6**	731 (28.1)
Age, median (IQR)	64.5 (53.6–75.1)
Female, n (%)	438 (59.9)
**PMS2**	233 (8.9)
Age, median (IQR)	66.1 (51.9–74.0)
Female, n (%)	131 (56.2)
**EPCAM**	23 (0.9)
Age, median (IQR)	65.0 (54.1–76.9)
Female, n (%)	16 (69.6)
**PDAC**, n (%)	17 (0.7)
**Suspected PDAC**, n (%)	5 (0.2)
**Ampullary carcinoma**, n (%)	10 (0.4)
**Distal cholangiocarcinoma**, n (%)	2 (0.1)

*Abbreviations*: IQR = interquartile range; LS = Lynch syndrome; n = number; PDAC = pancreatic ductal adenocarcinoma.

a One individual with pathogenic variants in both MLH1 and PMS2 was included in the MLH1 group.

### Cumulative incidence of PDAC, AC and dCC in Lynch syndrome carriers

The cumulative incidence of PDAC at age 75 in the total LS-cohort was 1.2% (95% CIs, 0.7–2.0). Subgroup-specific estimates at age 75 were 1.4% (95% CIs, 0.5–4.0) for *MLH1*, 2.1% (95% CIs, 1.0–4.1) for *MSH2*/*EpCAM*, 0.2% (95% CIs, 0.0–1.3) for *MSH6* and 0% for *PMS2*. When combining both PDAC cases and suspected PDAC cases, the cumulative incidence by age 75 was 1.5% (95% CIs, 0.9–2.4) in the total LS-cohort, 2.2% (95% CIs, 1.0–4.8) in the *MLH1* subgroup, 2.5% (95% CIs, 1.3–4.6) in *MSH2*/*EpCAM*, 0.2% (95% CIs, 0.0–1.3) in *MSH6* and 0% in *PMS2*. Among the 17 PDAC cases, none occurred within the same family.

All cumulative incidence estimates at ages 70, 75 and 80 are presented in Table 2 for confirmed PDAC, the combination of confirmed and suspected PDAC, AC, dCC and the total of all cancers combined. Estimates are shown both overall and stratified by genetic subgroup. Like PDAC, all identified AC and dCC cases occurred in separate families, with no familial-clustering observed.

**Table 2:** Cumulative incidence of pancreatic, ampullary and distal cholangiocarcinoma in individuals diagnosed with Lynch syndrome.

Cohort	Age	Cumulative incidence (95% CIs)				
		PDAC	PDAC + suspected	AC	dCC	Total^a^
Overall	70	0.6% (0.3–1.2)	0.9% (0.5–1.5)	0.2% (0.1–0.6)	0.2% (0.0–0.6)	1.2% (0.8–1.9)
	75	1.2% (0.7–2.0)	1.5% (0.9–2.4)	0.6% (0.3–1.3)	0.2% (0.0–0.6)	2.3% (1.5–3.3)
	80	1.6% (0.9–2.9)	2.0% (1.2–3.3)	1.3% (0.6–2.5)	0.2% (0.0–0.6)	3.4% (2.3–5.0)
MLH1	70	0.4% (0.1–1.8)	0.8% (0.3–2.2)	0%	0.3% (0.0–2.3)	1.1% (0.5–2.8)
	75	1.4% (0.5–4.0)	2.2% (1.0–4.8)	0.4% (0.1–3.1)	0.3% (0.0–2.3)	3.0% (1.5–5.8)
	80	1.4% (0.5–4.0)	2.2% (1.0–4.8)	0.4% (0.1–3.1)	0.3% (0.0–2.3)	3.0% (1.5–5.8)
MSH2/EPCAM	70	1.3% (0.6–2.8)	1.7% (0.9–3.4)	0.5% (0.1–1.6)	0%	2.2% (1.2–4.0)
	75	2.1% (1.0–4.1)	2.5% (1.3–4.6)	0.9% (0.3–2.7)	0%	3.4% (2.0–5.8)
	80	3.6% (1.8–7.3)	4% (2.1–7.7)	2.1% (0.8–5.3)	0%	6.2% (3.6–10.3)
MSH6	70	0.2% (0.0–1.3)	0.2% (0.0–1.3)	0.2% (0.0–1.5)	0.2% (0.0–1.4)	0.6% (0.2–1.9)
	75	0.2% (0.0–1.3)	0.2% (0.0–1.3)	0.6% (0.1–2.4)	0.2% (0.0–1.4)	1.0% (0.3–2.7)
	80	0.2% (0.0–1.3)	0.2% (0.0–1.3)	1.4% (0.4–4.9)	0.2% (0.0–1.4)	1.8% (0.6–5.0)
PMS2	70	0%	0%	0%	0%	0%
	75	0%	0%	0%	0%	0%
	80	0%	0%	0%	0%	0%

*Abbreviations:* AC = ampullary carcinoma; CIs = confidence intervals; dCC = distal cholangiocarcinoma; PDAC = pancreatic ductal adenocarcinoma.

a Total includes suspected PDACs.

### Relative risks of PDAC, AC and dCC in LS compared to sporadic cases in the general population

The cumulative incidences of sporadic cases of PDAC, AC, dCC and the combined total are shown in Supplemental Table S1. The cumulative incidence of sporadic PDAC in the general population at age 75 is estimated to be 0.7%.

For PDAC, the overall RR for the LS cohort by age 75 was 1.7 (95% CIs, 1.0–2.9). Subgroup-specific RRs by age 75 were 2.0 (95% CIs, 0.7–5.7) for *MLH1*, 3.0 (95% CIs, 1.5–6.1) for *MSH2*/*EpCAM*, 0.3 (95% CIs, 0.0–Inf) for *MSH6* and 0 for *PMS2*. When evaluating the combined outcome of PDAC and suspected PDAC, the RR for the entire LS cohort compared to the general population at age 75, was 2.1 (95% CIs, 1.3–3.5). The gene-specific RRs for this combined outcome were 3.1 (95% CIs, 1.4–6.9) for *MLH1*, 3.6 (95% CIs, 1.9–6.7) for *MSH2*/*EpCAM*, 0.3 (95% CIs, 0.0-Inf) for *MSH6* and 0 for *PMS2*. An overview of the RRs for the various cancers in individuals with LS compared to the general population is provided in Table 3.

**Table 3:** Relative risks of pancreatic, ampullary and distal cholangiocarcinoma in LS compared to the sporadic cases.

Cohort	Age	Relative risk				
		PDAC	PDAC + suspected	AC	dCC	Total
Overall	70	1.3 (0.7–2.7)	2.0 (1.2–3.5)	6.7 (2.7–16.7)	6.7 (0.0–Inf)	2.4 (1.6–3.7)
	75	1.7 (1.0–2.9)	2.1 (1.3–3.5)	15.0 (6.9–32.6)	5.0 (0.0–Inf)	2.9 (2.0–4.3)
	80	1.6 (0.9–2.8)	1.9 (1.2–3.2)	21.7 (10.4–45.1)	2.9 (0.0–Inf)	3.0 (2.0–4.4)
MLH1	70	0.9 (0.2–3.8)	1.8 (0.7–4.8)	0.0	10.0 (0.0–Inf)	2.2 (0.9–5.2)
	75	2.0 (0.7–5.7)	3.1 (1.4–6.9)	10.0 (1.8–56.7)	7.5 (0.0–Inf)	3.8 (1.9–7.5)
	80	1.4 (0.5–3.9)	2.1 (1.0–4.7)	6.7 (1.2–37.4)	4.3 (0.0–Inf)	2.6 (1.3–5.1)
MSH2/EPCAM	70	2.9 (1.3–6.3)	3.8 (1.9–7.4)	16.7 (4.1–67.7)	0.0	4.4 (2.4–8.1)
	75	3.0 (1.5–6.1)	3.6 (1.9–6.7)	22.5 (7.3–69.5)	0.0	4.3 (2.5–7.4)
	80	3.5 (1.7–7.1)	3.9 (2.0–7.5)	35.0 (13.4–91.4)	0.0	5.4 (3.2–9.1)
MSH6	70	0.4 (0.0–Inf)	0.4 (0.0–Inf)	6.7 (0.0–Inf)	6.7 (0.0–Inf)	1.2 (0.4–3.7)
	75	0.3 (0.0–Inf)	0.3 (0.0–Inf)	15.0 (3.0–75.0)	5.0 (0.0–Inf)	1.3 (0.4–3.8)
	80	0.2 (0.0–Inf)	0.2 (0.0–Inf)	23.3 (6.6–82.6)	2.9 (0.0–Inf)	1.6 (0.5–4.5)
PMS2^a^	70	0.0	0.0	0.0	0.0	0.0
	75	0.0	0.0	0.0	0.0	0.0
	80	0.0	0.0	0.0	0.0	0.0

*Abbreviations:* AC = ampullary carcinoma; CIs = confidence intervals; dCC = distal cholangiocarcinoma; LS = Lynch syndrome; PDAC = pancreatic ductal adenocarcinoma.

a No cases were observed in PMS2 carriers.

### Survival comparison of PDAC in LS and matched sporadic cases

The clinical details of individuals diagnosed with PDAC in the LS cohort and in the matched sporadic cases are presented in Supplemental Table S2. All PDACs developed in individuals previously diagnosed with LS; no index cases were identified. Among the seventeen PDAC cases within the LS cohort, two (11.8%) were stage I, one (5.9%) stage II, six (35.3%) stage III, seven (41.2%) stage IV and one case (5.9%) was of an unknown stage. With respect to the MMR status, three (17.6%) cases were classified as MMRd, one (5.9%) was MMR proficient and the remaining thirteen (76.5%) were not tested. The baseline characteristics of PDAC patients from the LS cohort and matched individuals from the general population are presented in Supplemental Table S3a.

**Fig. 1:**
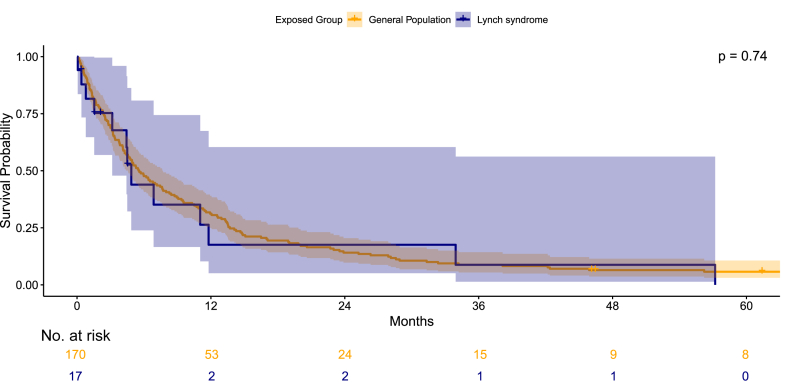
Kaplan–Meier showing five-year survival of pancreatic ductal adenocarcinoma in Lynch syndrome compared to the matched sporadic cases.

The overall survival outcomes for individuals with LS who developed PDAC were not significantly different (p = 0.74) from those observed in the matched individuals (see Fig. 1). The two-year survival rate for PDAC in the LS and the matched cohort was 17.6% (95% CIs, 5.1–60.4) and 14.1% (95% CIs, 9.7–20.5), respectively. The five-year survival could not be estimated for the LS cohort due to the absence of cases beyond five years of follow-up. The median overall survival was 4.9 months (95% CIs, 3.2–Inf) in the LS cohort and 5.6 months (95% CIs, 4.6–7.7) in the matched cohort.

### Survival comparison of ampullary carcinoma in Lynch syndrome and matched sporadic cases

Of the ten AC cases, one (10%) was stage I, four (40%) stage III, one (10%) stage IV and the remaining four (40%) were of an unknown stage. Regarding the MMR status, four (40%) individuals had MMRd tumors, while the remaining six (60%) were not tested. The clinical details of all individuals diagnosed with AC are provided in Supplemental Table S2 and the baseline characteristics of these patients and the matched individuals from the general population are presented in Supplemental Table S3b.

**Fig. 2:**
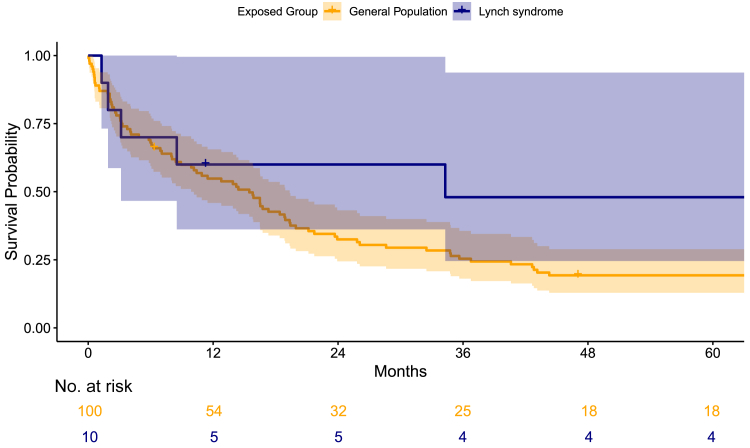
Kaplan–Meier showing five-year survival of ampullary carcinoma in Lynch syndrome compared to the matched sporadic cases.

Due to the low number of AC cases, we refrained from performing a log-rank test. Nevertheless, the observed survival curves suggest a trend toward differences between individuals with LS who developed AC and the matched individuals (see Fig. 2). The two-year survival for AC in the LS cohort and the matched cohort was 60% (95% CIs, 36.2–99.5) and 32.5% (95% CIs, 24.5–43.2), respectively. The five-year survival was 48% (95% CIs, 24.6–93.8) in the LS cohort and 19.3% (95% CIs, 12.9–28.9) in the matched cohort. The median survival was 34.3 months (95% CIs, 3.2–Inf) for individuals with LS, as compared to 15.5 months (95% CIs, 9.9–19.3) for the matched individuals.

### Survival comparison of distal cholangiocarcinoma in Lynch syndrome and matched sporadic cases

Due to an insufficient number of cases, survival analysis for dCC was not performed.

## Discussion

This is the first nationwide prospective cohort study in individuals with LS to evaluate the cumulative incidence of cancers potentially detectable through image-based PDAC surveillance. Our findings show that none of the carriers of PVs in LS-associated genes reached the 5% risk threshold as advised by CAPS for PDAC surveillance, even when PDAC, AC and dCC were combined.^6^ By age 75, the estimated combined cumulative incidence was 3.0% (95% CIs, 1.5–5.8) for *MLH1* carriers, 3.4% (95% CIs, 2.0–5.8) for *MSH2*/*EpCAM*, 1.0% (95% CIs, 0.3–2.7) for *MSH6* and 0% for *PMS2* carriers. Notably, although *MLH1* (RR: 3.8; 95% CIs, 1.9–7.5) and *MSH2/EPCAM* carriers (RR: 4.3; 95% CIs, 2.5–7.4) demonstrate an increased RR of PDAC compared with the general population, these increases did not translate into reaching the absolute risk threshold required to justify surveillance. Furthermore, none of the PDAC cases occurred within the same family, providing no evidence that having an affected relative substantially increases risk, though interpretation is limited by small numbers. These findings suggest that the current recommendation to offer surveillance to individuals with LS based on a single affected relative may warrant re-evaluation.

To date, no studies have specifically examined the risk of AC and dCC in individuals with LS, as these malignancies are typically grouped within broader gastrointestinal cancer categories, limiting comparability. However, four studies have investigated the age-specific cumulative incidence of PDAC in LS. Barrow et al.^3^ studied 839 individuals with LS in North West of England, reporting cumulative incidences at age 70 of 0% for *MLH1,* 0.7% (95% CIs, 0.0–1.4) for *MSH2* and 0% for *MSH6*.^3^ These findings are comparable with our results (0.4% for *MLH1*, 1.3% for *MSH2*/*EpCAM* and 0.2% for *MSH6* at age 70), although the low incidence in their study may reflect broader inclusion of individuals without confirmed PVs. In contrast, Kastrinos et al.^4^ reported a notably higher PDAC incidence of 3.7% (95% CIs, 1.5–5.9) at age 70 in a multicenter cohort of 6342 LS carriers, likely influenced by the overrepresentation of *MLH1*and *MSH2* PV carriers (93%), with 44 of 47 PDAC cases occurring in these subgroups. Furthermore, the potential misclassification resulting from the absence of pathological or clinical data in 78.7% of cases may have contributed to the overestimation of the incidence. A more recent study by Møller et al.^5^ reported on an international cohort of 3119 LS individuals, finding cumulative PDAC incidence at age 70 of 3.9% (95% CIs, 1.4–6.4) for *MLH1*, 0.5% (95% CIs, 0.0–1.5) for *MSH2*, 1.4% (95% CIs, 0.0–4.2) for *MSH6* and 0% for *PMS2*. Although these estimates appear higher than in our study, the 95% CIs overlap, indicating that the findings are consistent with our results. The wider intervals reported by Møller et al.^5^ suggest that our estimates are more precise, likely due to the older median age at last follow-up in our cohort, despite our cohort still being relatively young overall. The most recent study by Bujanda et al.^17^ reported cumulative incidence at age 70 of 3.3% (95% CIs, 0.0–7.0) for *MLH1*, 2.6% (0.0–7.7) for *MSH2/*EPCAM and 0% for *MSH6* and *PMS2*. Similarly, although the point estimates are somewhat higher, the overlapping 95% CIs indicate that these findings are compatible with our results. A complete overview of published cumulative incidence estimates is provided in Supplemental Table S4.

In the LS cohort, PDAC survival was comparable to that of matched sporadic cases. In contrast, AC survival appeared more favorable in LS, although the small number warrants cautious interpretation. Our findings of no PDAC survival benefit in the LS cohort align with a recent meta-analysis that also found no advantage in MMRd PDAC.^18^ Nevertheless, the authors of that study emphasized that the evidence remains limited and inconclusive.^18^ Prior studies have reported an association between MMRd and improved prognosis, as well as responsiveness to immune checkpoint blockade (ICB).^19^^,^^20^ Notably, one series of resected PDACs reported a 5-year overall survival of 77% in MSI-H versus 27% in microsatellite stable tumors.^20^ In contrast, the potential benefit may not have been detectable in our cohort due to several mitigating factors. These include the high proportion of advanced-stage diagnoses (41.2% of cases), absence of ICB therapy and the possibility that some tumors were not MSI-H/MMRd. While ICBs have not shown to improve outcomes in sporadic PDAC, its role in MSI-H/MMRd PDAC remains under investigation.^21^, ^22^, ^23^, ^24^ These findings underscore the need for prospective clinical trials and highlight the importance of identifying MSI-H/MMRd PDAC and stratifying this subgroup in survival analyses.

Although the prevalence of MMRd in sporadic PDAC is low (<1%), the National Comprehensive Cancer Network (NCCN) recommends comprehensive tumor gene profiling, including MMRd assessment, for all patients with locally advanced or metastatic PDAC.^25^, ^26^, ^27^ The European Society for Medical Oncology (ESMO) recommends MMRd testing in sporadic PDAC specifically as a predictor for ICB, using next-generation sequencing.^28^ In contrast, the Dutch guideline recommends MMR testing for all PDACs diagnosed under the age of 50 years.^29^ Beyond these recommendations, individuals with LS have a higher likelihood of harboring MSI-H/MMRd tumors.^25^^,^^26^ Among 824 PDACs, 34 were identified as MSI-H/MMRd, of which 5 (14.7%) were attributed to LS, underscoring the relevance of testing in this population.^19^ In our LS cohort, 76% of PDAC cases were not tested for MSI/MMRd status, reflecting either non-adherence to current international guidelines or the absence of intent to pursue ICB therapy in these individuals.

One limitation of this study is the lack of adjustment for lifestyle factors, such as smoking, alcohol consumption and physical activity, leaving the potential influence of these variables on PDAC development unaccounted for. Another limitation is the relatively young age of the cohort, with a median age of 64 years at the end of the study. This makes the cumulative incidence estimates at older ages less precise, as reflected by the wider CIs. Additionally, there is a risk of misclassification bias due to the inclusion of pathologically unconfirmed PDAC cases. A substantial proportion of PDAC diagnoses lack histological confirmation, as many patients are diagnosed at an advanced stage where biopsy is not pursued because it would not alter clinical management. However, excluding clinically confirmed but pathologically unconfirmed cases would conversely lead to an underestimation of PDAC incidence. As this is a retrospective study, some variability in diagnostic practices over time may also have contributed to limited misclassification. These limitations apply to both the LS cohort and sporadic cases. Moreover, our survival analyses were limited by missing MSI-H/MMRd status data. Without this information, we were unable to evaluate how MMRd PDACs would perform. Furthermore, although recruitment to StOET is nationwide and ongoing, the overall coverage of LS mutation carriers in the Netherlands is unknown. Lastly, the definition of sporadic cases in the general population is not well defined. While our LS cohort was compared to the general population data provided by the NCR, this group also includes individuals with an increased genetic risk of PDAC, such as carriers of *CDKN2A* or *BRCA* PVs. Due to privacy restrictions, it was not possible to identify and exclude these high-risk individuals. As a result, the RR of PDAC in LS carriers may have been underestimated when compared to truly sporadic cases.

In conclusion, our findings demonstrate that none of the LS-associated genes exhibit a combined cumulative incidence of PDAC, AC, and dCC (ranging from 0 to 3.4% depending on the gene) that exceeds the currently suggested threshold for initiating pancreatic surveillance. Furthermore, there was no compelling evidence of increased familial PDAC risk in our cohort. Finally, survival was comparable between LS and matched sporadic cohorts, except for AC where outcomes were more favorable in LS.

## Contributors

**Conceptualisation:** A.M.B, D.C.F.K, M.E.L **Data curation:** A.M.B, L.A.A.B, L.G.G, M.E.L **Formal analysis:** A.M.B, M.E.L **Accessed and verified the data:** A.M.B, M.E.L **Funding acquisition:** A.M.B, M.E.L, J.E.H, D.C.F.K **Investigation:** M.E.L, J.E.H **Methodology:** A.M.B, D.C.F.K, M.E.L **Project administration:** A.M.B, D.C.F.K, M.E.L, J.E.H, L.G.G **Resources:** B.A.B, L.A.A.B, E.D, L.G.G, J.E.G.I, J.J.K, M.C.A.K, A.M.J.L, M.N, D.R, M.C.S, W.H.V.N.C, J.E.H, M.E.L **Software:** - **Supervision:** D.C.F.K, M.E.L, J.E.H **Validation:** A.M.B, D.C.F.K, M.E.L, L.A.A.B, L.G.G **Visualisation:** A.M.B **Writing (original draft):** A.M.B **Writing (review & editing):** All authors contributed equally.

## Data sharing statement

Deidentified participant data will be made available upon reasonable request to the corresponding author. Access will be granted for academic, non-commercial purposes following approval of a proposal and completion of a data sharing agreement. Data will be shared beginning with publication.

## Declaration of interests

All authors declare no conflicts of interest.
