# Supplementary material for: Pancreatic cancer risk and survival in patients with Lynch syndrome: a nationwide Dutch cohort study
Source: eClinicalMedicine. 2026 Jan 12;91:103755. doi: 10.1016/j.eclinm.2026.103755 (PMC12828513; doi:10.1016/j.eclinm.2026.103755)
Supplement: Supplementary Tables [file mmc1.docx]

**SUPPLEMENTAL MATERIALS**

**PANCREATIC CANCER RISK AND SURVIVAL IN PATIENTS WITH LYNCH SYNDROME: A NATIONWIDE DUTCH COHORT STUDY**

**Contents**

[**List of collaborator group individuals** 2](#_Toc216858857)

[**Supplemental Table 1. Cumulative incidence of pancreatic, ampullary and distal cholangiocarcinoma in the general population** 3](#_Toc216858858)

[**Supplemental Table 2. Clinical details of Lynch syndrome individuals diagnosed with (suspected) pancreatic, ampullary and distal cholangiocarcinoma** 4](#_Toc216858859)

[**Supplemental Table 3a.** **Baseline characteristics of pancreatic ductal adenocarcinoma in Lynch syndrome and matched sporadic cases** 6](#_Toc216858860)

[**Supplemental Table 3b. Baseline characteristics of ampullary carcinoma in Lynch syndrome and matched sporadic cases** 7](#_Toc216858861)

[**Supplemental Table 4.** **Summary of existing evidence** 8](#_Toc216858862)

# **List of collaborator group individuals**

We thank all individuals registered at the Netherlands Foundation for Detection of Hereditary Tumors and all collaborative investigators of the Netherlands Foundation for Detection of Hereditary Tumors registry: A.A. Tanis; A.Y. Thijssen; W.R. ten Hove; J. Sint Nicolaas; F. Voogd; N.C. Talstra; M.J. van Heerde; C.C.G. van Enckevort; E.J. Schoon; C. Postma; A. Geraedts; F. ter Borg; J. Geesing; M.G.F. van Lier; W. Hazen; M.L. Hazen; M.W. Mundt; I. Leeuwenburgh; G.W. Erkelens; M. Kerkhof; J.J. Keller; M.H.M.G. Houben; J.P. De Filippi; T.J. Tang; C. Verveer; J.S. Terhaar sive Droste; M.W. van den Berg; P.J. Bus; J.J.L. Haans; W.J. Thijs; M.L. Verhulst; L.G. Capelle; S. Corporaal; M. Bigirwamungu-Bargeman; A.M. Zonneveld; A.M. van Berkel; W.E. Boertien; A.U.G. van Lent; S.A. Mulder; F.A. Oort; M.I.E. Appels; R. Andriessen; W.A. Marsman; S. de Kort; A. Al-Toma; M. van Boekel; M.E. Smits; P.E.P. Dekkers; T. Kuiper; J.E. van Rooij; R. Meiland; L. van Vlerken; H. Aktas and E. Rondagh.

# **Supplemental Table 1. Cumulative incidence of pancreatic, ampullary and distal cholangiocarcinoma in the general population**

|  |  | **Cumulative incidence** (95% CIs) | | | |
| --- | --- | --- | --- | --- | --- |
| **Cohort** | **Age** | **PDAC** | **AC** | **dCC** | **Total** |
| General population | 70 | 0.45% (0.42-0.48) | 0.03% (0.02-0.03) | 0.03% (0.02-0.03) | 0.50% (0.47-0.53) |
|  | 75 | 0.70% (0.66-0.74) | 0.04% (0.03-0.05) | 0.04% (0.03-0.05) | 0.79% (0.74-0.83) |
|  | 80 | 1.03% (0.97-1.08) | 0.06% (0.05-0.07) | 0.07% (0.05-0.08) | 1.15% (1.10-1.21) |

*Abbreviations:* AC= ampullary carcinoma; CIs= confidence intervals; dCC= distal cholangiocarcinoma; PDAC= pancreatic ductal adenocarcinoma.

# **Supplemental Table 2. Clinical details of Lynch syndrome individuals diagnosed with (suspected) pancreatic, ampullary and distal cholangiocarcinoma**

| **No.** | **Type of cancer** | **Age at Diagnosis** | **Year of diagnosis (bracket)** | **Sex** | **Stage** | **IHC staining absence** | **Follow-up** (months)* | **Gene** | **Diagnosed based on** |
| --- | --- | --- | --- | --- | --- | --- | --- | --- | --- |
| P1 | PDAC | 61 | 2021–2025 | F | IV | MLH1 | 4 | MLH1 | Pathology |
| P2 | PDAC | 80 | 2021–2025 | M | IV | Unknown | 2 | MSH6 | Imaging |
| P3 | PDAC | 62 | 2021–2025 | M | III | No | 1 | MSH2 | Pathology |
| P4 | PDAC | 57 | 2011–2015 | F | IV | Unknown | 3** | MSH2 | Pathology |
| P5 | PDAC | 73 | 2011–2015 | M | IV | Unknown | 0** | MLH1 | Pathology |
| P6 | PDAC | 72 | 2011–2015 | M | III | Unknown | 6** | MSH2 | Pathology |
| P7 | PDAC | 79 | 2021–2025 | M | III | MSH2 | 0 | MSH2 | Pathology |
| P8 | PDAC | 63 | 2006–2010 | F | Unknown | Unknown | 1** | MSH2 | Pathology |
| P9 | PDAC | 74 | 2016–2020 | F | II | Unknown | 1** | MLH1 | Pathology |
| P10 | PDAC | 56 | 1996–2000 | F | III | Unknown | 0** | MSH2 | Pathology |
| P11 | PDAC | 65 | 2001–2005 | F | IV | Unknown | 4** | EPCAM | Pathology |
| P12 | PDAC | 56 | 2011–2015 | M | IV | Unknown | 10** | MSH2 | Imaging |
| P13 | PDAC | 78 | 2016–2020 | F | III | Unknown | 30** | MSH2 | Pathology |
| P14 | PDAC | 54 | 2011–2015 | M | IV | Unknown | 4** | MSH6 | Imaging |
| P15 | PDAC | 58 | 2011–2015 | M | I | Unknown | 10** | MLH1 | Pathology |
| P16 | PDAC | 70 | 2001–2005 | M | III | Unknown | 4** | MSH2 | Pathology |
| P17 | PDAC | 60 | 2011–2015 | M | I | MSH2 and MSH6 | 51** | MSH2 | Pathology |
| P18 | Suspected PDAC | 46 | 2016–2020 | M | Unknown | Unknown | 2** | MLH1 | Pathology |
| P19 | Suspected PDAC | 46 | 2016–2020 | F | Unknown | Unknown | 66 | MSH2 | Pathology |
| P20 | Suspected PDAC | 67 | 2021–2025 | F | IV | Unknown | 0 | MSH2 | Pathology |
| P21 | Suspected PDAC | 71 | 2016–2020 | F | II | Unknown | 87 | MLH1 | Pathology |
| P22 | Suspected PDAC | 59 | 1990–1995 | F | Unknown | Unknown | 239** | MLH1 | Pathology |
| P23 | AC | 70 | 2001–2005 | M | Unknown | MSH6 | 251 | MSH6 | Pathology |
| P24 | AC | 32 | 1996–2000 | F | III | Unknown | 333 | MSH2 | Pathology |
| P25 | AC | 77 | 2016–2020 | F | Unknown | Unknown | 1** | MSH2 | Pathology |
| P26 | AC | 59 | 2021–2025 | M | III | MSH6 | 11 | MSH6 | Pathology |
| P27 | AC | 73 | 2011–2015 | M | Unknown | Unknown | 3** | MLH1 | Pathology |
| P28 | AC | 65 | 2011–2015 | M | I | MSH2 | 152 | MSH2 | Pathology |
| P29 | AC | 76 | 2011–2015 | F | III | Unknown | 2** | MSH2 | Pathology |
| P30 | AC | 72 | 2021–2025 | M | IV | Unknown | 9** | MSH2 | Pathology |
| P31 | AC | 79 | 2011–2015 | F | Unknown | Unknown | 34** | MSH6 | Pathology |
| P32 | AC | 53 | 2001–2005 | F | III | MSH2 and MSH6 | 219** | MSH2 | Pathology |
| P33 | dCC | 68 | 2016–2020 | F | II | Unknown | 100 | MLH1 | Pathology |
| P34 | dCC | 57 | 2011–2015 | M | II | Unknown | 9** | MSH6 | Pathology |

*Abbreviations*: *= Time interval from the date of diagnosis until death or last available follow-up, expressed in months; **= deceased; AC= ampullary carcinoma; dCC= distal cholangiocarcinoma; F= female; IHC= immunohistochemistry; M= male; MMR= mismatch repair; PDAC= pancreatic ductal adenocarcinoma.

# **Supplemental Table 3a.** **Baseline characteristics of pancreatic ductal adenocarcinoma in Lynch syndrome and matched sporadic cases**

|  | PDAC cases in Lynch syndrome cohort (N=17) | Matched sporadic cases in general population  (N= 170) |
| --- | --- | --- |
| Age at diagnosis,  median (IQR) | 63.0 (58.0-73.0) | 63.0 (58.0-73.0) |
| Sex, female, n (%) | 7 (41.2) | 70 (41.2) |
| Stage, n (%) |  |  |
| I | 2 (11.8) | 20 (11.8) |
| II | 1 (5.9) | 10 (5.9) |
| III | 6 (35.3) | 60 (35.3) |
| IV | 7 (41.2) | 70 (41.2) |
| Unknown | 1 (5.9) | 10 (5.9) |
| Year of diagnosis, median (IQR) | 2015 (2011-2020) | 2015 (2011-2020) |

*Abbreviations*: IQR= interquartile range; n= number; PDAC= pancreatic ductal adenocarcinoma.

# **Supplemental Table 3b. Baseline characteristics of ampullary carcinoma in Lynch syndrome and matched sporadic cases**

|  | AC cases in Lynch syndrome cohort (N=10) | Matched sporadic AC cases in general population  (N= 100) |
| --- | --- | --- |
| Age at diagnosis,  median (IQR) | 71.0 (60.5-75.3) | 70.0 (57.0-76.0) |
| Sex, female, n (%) | 5 (50.0) | 50 (50.0) |
| Stage, n (%) |  |  |
| I | 1 (10.0) | 10 (10.0) |
| II | 0 | 0 |
| III | 4 (40.0) | 40 (40.0) |
| IV | 1 (10.0 | 10 (10.0) |
| Unknown | 4 (40.0) | 40 (40.0) |
| Year of diagnosis, median (IQR) | 2012 (2006-2018) | 2012 (2005-2017) |

*Abbreviations*: AC= ampullary carcinoma; IQR= interquartile range; n= number.

# **Supplemental Table 4.** **Summary of existing evidence**

|  |  | **Cumulative incidence by age 70 (95% CIs)** | | | |
| --- | --- | --- | --- | --- | --- |
| **Study** | **Sample size** | **MLH1** | **MSH2/EPCAM** | **MSH6** | **PMS2** |
| Barrow et al. 2009 | 839 | 0% | 0.7%  (0.0-1.4) | 0% | – |
| Kastrinos et al. 2009 | 6342 | 3.7% (1.5-5.9) in the overall cohort | | | |
| Møller et al. 2017 | 3119 | 3.9%  (1.4-6.4) | 0.5%  (0.0-1.5) | 1.4%  (0.0-4.2) | 0% |
| Bujanda et al. 2025 | 425 | 3.3%  (0.0-7.0) | 2.6%  (0.0-7.7) | 0% | 0% |
| Our study | 2605 | 0.4%  (0.1-1.8) | 1.3%  (0.6-2.8) | 0.2%  (0.0-1.3) | 0% |

- = not evaluated
